# Slow conduction and gap junction remodeling in murine ventricle after chronic alcohol ingestion

**DOI:** 10.1186/1423-0127-18-72

**Published:** 2011-09-29

**Authors:** Yu-Jun Lai, Chung-Lieh Hung, Ray-Ching Hong, Ya-Ming Tseng, Cheng-I Lin, Yu-Shien Ko, Cheng-Ho Tsai, Hung-I Yeh

**Affiliations:** 1 Departments of Internal Medicine and Medical Research, Mackay Memorial Hospital, Mackay Medicine, Nursing and Management College, Mackay Medical College, New Taipei City, Taiwan; 2 Departments of Physiology and Biophysics, National Defense Medical Center, Taipei, Taiwan; 3 The First Cardiovascular Division, Department of Internal Medicine, Chang Gung Memorial Hospital, Taipei, Taiwan

**Keywords:** alcohol, arrhythmia, remodeling, gap junctions, optical mapping

## Abstract

**Background:**

Long-term heavy alcohol drinkers are prone to the development of cardiac arrhythmia. To understand the mechanisms, we evaluated the cardiac structural and electrophysiological changes in mice chronically drinking excessive alcohol.

**Results:**

Male C57BL/6J mice were given 36% alcohol in the drinking water. Those given blank water were used as control. Twelve weeks later, the phenotypic characteristics of the heart, including gap junctions and electrical properties were examined. In the alcohol group the ventricles contained a smaller size of cardiomyocytes and a higher density of capillary networks, compared to the control. Western blots showed that, after drinking alcohol, the content of connexin43 (Cx43) protein in the left ventricle was increased by 18% (p < 0.05). Consistently, immunoconfocal microscopy demonstrated that Cx43 gap junctions were up-regulated in the alcohol group with a disorganized distribution, compared to the control. Optical mapping showed that the alcohol group had a reduced conduction velocity (40 ± 18 vs 60 ± 7 cm/sec, p < 0.05) and a higher incidence of ventricular tachyarrhythmia (62% vs 30%, p < 0.05).

**Conclusion:**

Long-term excessive alcohol intake resulted in extensive cardiac remodeling, including changes in expression and distribution of gap junctions, growth of capillary network, reduction of cardiomyocyte size, and decrease of myocardial conduction.

## Background

Excessive alcohol ingestion is harmful to the heart [1-4]. Previous studies have shown that manifestations of alcoholic cardiac suppression include mechanical dysfunction and electrical instability [5-7]. Physiologically, effective pumping of the heart requires coordination of contraction between individual cardiomyocytes, which depends mainly on the proper propagation of action potentials. Disturbance in the spread of action potential along the myocardium also plays a key role in the formation of cardiac arrhythmia. At the subcellular level, transmission of action potential between adjacent cardiomyocytes goes through gap junctions [8,9].

Gap junctions, composed of molecules belonging to the connexin multi-gene family, are clusters of cell membrane protein channels, which in the ventricular working cardiomyocytes are mainly made of connexin 43 (Cx43) [8,9]. Change of the expression patterns of the connexins has been demonstrated to be associated with mechanical dysfunction and contributes to the development of cardiac arrhythmia [10,11]. However, the effect of alcohol on the expression of cardiac connexins remained unclear. To this end we in this study examined the ventricular myocardium, including the morphology, the gap junction distribution, and connexins expression as well as the electrophysiological properties, in mice after 12-week intake of 36% alcohol as the only source of fluid. A previous study giving mice high concentration of ethanol in the drinking water showed that the blood level of ethanol in the animals reached the level affecting physiology and/or behavior [12].

## Results

### Weight change, alcohol levels, and histological examination

Initially the two groups of animals had similar weights (alcohol, 20.7 ± 0.2 g; control, 20.9 ± 0.2 g). However, one week later, in contrast to the weight gain in the control group (23.7 ± 0.2 g), the alcohol group decreased slightly in weight (20.1 ± 0.3 g). Thereafter both groups gained weight gradually and throughout the remaining experiment period the alcohol group was lighter than the control group (at the end of the experiment, 26.0 ± 1 vs 29.4 ± 0.8 g, p < 0.01). Similarly, at the end of the experiment the heart was lighter in the alcohol group (112 ± 3 vs 131 ± 3 mg, p < 0.01). Comparison of the ratio of heart weight to body weight showed that both groups had a similar ratio (alcohol 0.44 ± 0.01%, control 0.45 ± 0.01%). Alcohol was detected in the serum of the alcohol group (125 ± 13 mg/dl), but not detectable in the control group.

**Figure 1:**
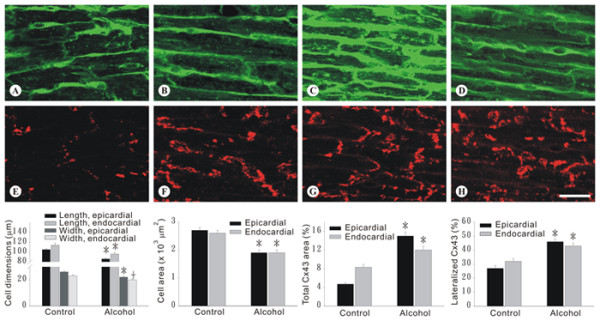
**Reduced cardiomyocyte size and disturbed gap junction distribution in the left myocardium of mice with long-term alcohol ingestion**. Immunocofocal images of cardiac muscle double stained for WGA (A through D) and Cx43 (E through H). Images in each column are of the same field. Compared to the control group (A and B) the cardiomyocytes in the alcohol group (C and D) are smaller in size, but contain more Cx43 gap junctions, as shown in the below histograms, each of which represents more than 80 randomly selected cells from three animals of each group. A and C are taken from the epicardial side, B and D from the endocardial side of the left ventricular free wall. Note that the labels of WGA are more prominent in the alcohol group. In each histograms * and + respectively indicate p < 0.01 and p < 0.05 compared to the corresponding bar of the control group. See text for details. All images are of the same magnification. Bar, 30 μm.

**Figure 2:**
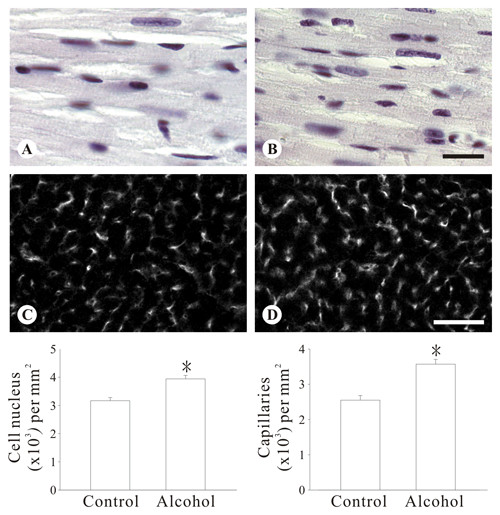
**Increased cellularity number and capillary network in the left ventricular myocardium of mice with long-term alcohol ingestion**. Cellularity (A and B, stained with hematoxyllin) and capillary network (C and D, stained for CD31) in the left ventricular myocardium. A and C, are from the control group and B and D from the alcohol group. Results of analysis are shown in the histograms. In each histogram (n = 4 or 5) * indicate p < 0.05 compared to the control group. See text for details. Images A and B are of the same magnification; C and D are of the same magnification. Bar in B, 20 μm; in D, 60 μm.

Histological examination showed a remarkable remodeling of ventricular cardiomyocytes after 12 weeks of alcohol drinking. In samples stained with WGA the cell borders were clearly seen (Figure 1). Analysis of cardiomyocytes lying horizontally in the sections showed that the cells were smaller in the alcohol group, and the difference of size was seen across the whole layer of the ventricular wall (length, epicardial, 86 ± 2 vs 105 ± 2 μm; endocardial 96 ± 3 vs 114 ± 4 μm; width, epicardial, 22 ± 1 vs 26 ± 1 μm; endocardial, 20 ± 1 vs 23 ± 1 μm; area, epicardial, 1.9 ± 0.1 vs 2.7 ± 0.1 × 10^3 ^μm^2^; endocardial, 1.9 ± 0.1 vs 2.6 ± 0.1 × 10^3 ^μm^2^; all p < 0.05, see Figure 1A through 1D). Consistently, the number of cell nucleus per unit area of cardiac muscle was increased in the alcohol group (3.2 ± 0.1 vs 3.9 ± 0.1 nuclei × 10^3^/mm^2^, p < 0.05, see Figure 2A, B, and left lower histogram). Apart from the change of the cardiomyocytes, a higher density of capillary network was seen in the alcohol group (3.6 ± 1.4 vs 2.5 ± 1.3 capillaries × 10^3^/mm^2^, p < 0.05, Figure 2C, D, and right lower histogram). Similarly, development of fibrosis was rarely found in the two groups.

### Immunodetection of Cx43 gap junctions

**Figure 3:**
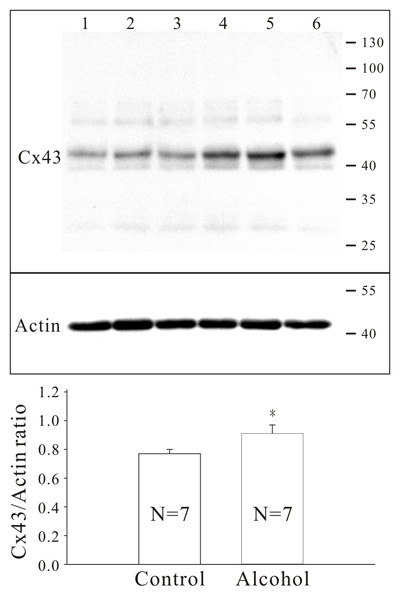
**Up-regulation of Cx43 protein in the left myocardium of mice after long-term ingestion of 36% alcohol**. Upper, an example of blots. Lanes 1-3, control group. Lanes 4-6, alcohol group. Results of denistometric analysis are shown in the lower histogram. The number of animals used for analysis is 7 in each group. * indicate p < 0.05 compared to the control group. See text for details.

Western blotting showed that the content of Cx43 protein in the left ventricle of the alcohol group was 18% more compared to the control (p < 0.05, see Figure 3). Such an up-regulation of Cx43 was also confirmed by immunoconfocal microscopy (Figure 1E through 1H), which, after image analysis, showed that in the alcohol group the amount of Cx43 gap junction area per cell was increased by 219.1% (p < 0.01) in the epicardial portion and 44.6% (p < 0.01) in the endocardial portion of the myocardium. In addition, the distribution of Cx43 gap junctions was altered. In the control group, Cx43 gap junctions were mainly confined to the intercalated disk area (Figure 1E and 1F). However, in mice drinking alcohol, a substantial amount of the gap junctions were present along the lateral border of the cardiomyocytes (epicardial, 46 ± 2 vs 27 ± 2%; endocardial, 43 ± 2 vs 32 ± 2%, both p < 0.05, see Figure 1G and 1H).

### Electron microscopy

**Figure 4:**
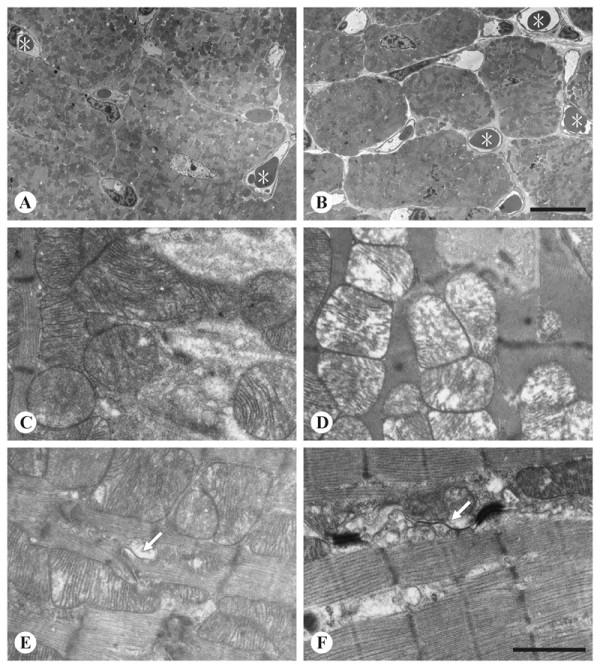
**Ultrastructural examination of intercardiomyocyte space, mitochondria, and gap junctions**. Ultrastructural examination of intercardiomyocyte space (A and B), mitochondria (C and D), and gap junctions (E and F). A, C, and E are from the control mice and B, D, and F from the alcoholic mice. See text for details. Note that compared to the control (A), in mice drinking alcohol (B) not only capillaries with loose pericapillary spaces (*) are more frequently seen, but the individual cardiomyocytes are smaller. Arrows in E and F indicate gap junctions. Images A and B are of the same magnification; C through F are of the same magnification. Bar in B, 10 μm; in F, 1 μm.

In the control group, cardiomyocytes were intimately packed together (Figure 4A). In contrast, in the alcohol group individual cardiomyocytes were loosely packed, owing to an increased number of capillaries as well as loose pericapillary spaces (Figure 4B). In addition to the extracellular difference, inside the cardiomyocytes swollen mitochondria with disrupted cistern were not infrequently seen in the alcohol group (Figure 4D), but rarely seen in the control (Figure 4C and 4E). Gap junctions were frequently seen in the vicinity of the adherens junctions for both groups (Figure 4E and 4F).

### Optical mapping

**Figure 5:**
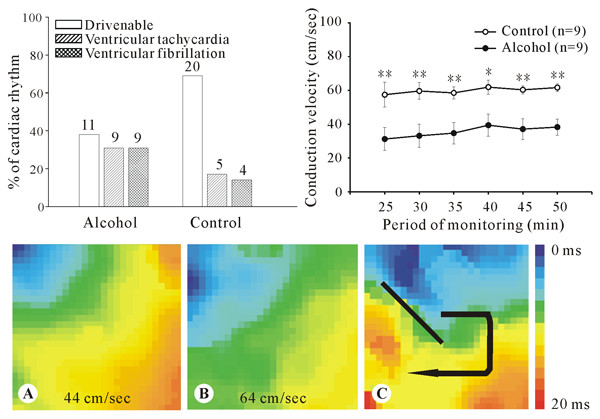
**Cardioelectrophysiologic studies of isolated hearts**. Upper left histogram shows the percentage of hearts drivenalbe by the pacemaker or manifesting ventricular arrhythmia. The number of animals is indicated at the top of each bar. Upper right histogram shows the values of conduction velocity measured at each time point of both groups. *, p < 0.05 and **, p < 0.01, compared to the control group. Lower panel, examples of activation maps with normal activation sequence from the alcohol (A) and the control group (B), as well as with conduction disturbance (C) from the alcohol group. The black bar means start of the reentry, and the curved arrow indicated the propagation direction Note that the values of conduction velocity in A and B are displayed at the bottom of the maps.

During optical mapping examination ventricular tachyarrhythmia, including ventricular tachycardia and ventricular fibrillation, was frequently found in the alcohol group. Therefore, we compared the incidence of ventricular tachyarrhythmia in 29 mice of each group. As shown in the upper left histogram of Figure 5, 18 (62%) mice of the alcohol group had ventricular tachyarrhythmia, in contrast to 9 (31%) mice in the control group (p < 0.05). In the remaining 11 mice of the alcohol group, 9 mice remained drivenable throughout the 50 minutes recording period. Conduction velocity, derived from activation map (Figure 5, lower panel), was calculated from the 9 mice of the alcohol group and compared to the initial 9 mice drivenable throughout the recording period of the control group. The result showed that the conduction velocity was significantly slower in the alcohol group (for each time point measured, all p < 0.05, see Figure 5A, B, and upper right histogram). In addition, in the activation map of the pacemaker-drivenable hearts the direction of activation in the control group was all from the left upper toward the right lower throughout the examination (Figure 5B). In contrast, conduction disturbance was commonly seen in the alcohol group (Figure 5C).

## Discussion

This study showed that, after ingesting 36% alcohol as the only source of fluid for 12 weeks, the murine ventricle underwent a remarkable remodeling, including reduction of cardiomyocyte size, increase in capillary network, up-regulation and lateralization of gap junctions, and slow down of conduction. In addition, animals in the alcohol group were more vulnerable to the development of ventricular tachyarrhythmia during optical mapping examination. These findings are complementary to the current knowledge of alcoholic heart disease.

In humans, long-term heavy alcohol consumption induces heart failure, the pathological findings of which are characterized by a form of non-ischemic, dilated cardiomyopathy, specifically called alcoholic cardiomyopathy [13]. Clinical studies of people regularly consuming large amount of alcohol have shown that before the development of heart failure the left ventricle volume increases and the ventricular wall thickness also increases [14,15]. Several controlled clinical studies have shown a dose-dependent depressor effect of alcohol on LV function, an effect that progressively induces the development of low-output dilated cardiomyopathy leading to congestive heart failure and sudden death [13,16,17]. However, information of the individual cardiomyocytes at an earlier stage before the heart is enlarged remained deficient. In the present study, compared to the control animals, the alcoholic animals had smaller cardiomyocytes, which is consistent with the grossly lighter hearts. The change of cell size is opposite to the finding from experiments feeding mice an alcohol-containing liquid diet, in which hypertrophy of cardiomyocytes was reported [18]. Chronic alcohol exposure had been reported to lead to a loss of myofibrillar protein [19], which may explain the reduction in cell size found in the present study. On the other hand, the absence of cardiac fibrosis in the present study is consistent with the previous report [18].

The increase in cell nucleus number per unit area in the present study is consistent with the smaller size of individual cardiomyocytes. However, the increase of nucleus is also attributable to the growth of capillary network, a novel finding of the present study. Previous studies in humans and rabbits had demonstrated that alcohol changed the morphology of capillary endothelial cells in the heart [20,21]. To our knowledge the growth of capillary network in the heart after chronic excessive alcohol consumption had not been reported. Regarding the mechanism of the growth, although moderate levels of ethanol was shown to induce expression of vascular endothelial growth factor and stimulate angiogenesis [22], chronic ingestion of ethanol in rats was reported to increase the ventricular expression of p53 [23], which is known to inhibit angiogenesis [24]. Exploration of the mechanisms underlying the growth of capillary network found in the present study requires further studies.

Another novel finding in the present study is that alcohol induced remodeling of gap junction in the cardiomyocytes, including up-regulation of the total amount of Cx43 protein (as demonstrated by Western blotting) and redistribution of Cx43 gap junctions at the cell membrane (confirmed by immunoconfocal microscopy). One might wonder in the present study why the increment of gap junction shown in Western blotting is much less compared to the immunoconfocal results. This is because in the immunoconfocal experiments the expression of gap junctions was calibrated against cell size, which was reduced substantially in the alcohol group. On the other hand, theoretically, more gap junctions in the cardiac muscle should facilitate the electric conduction [25]. In addition, more gap junctions in the lateral borders of cardiomyocytes should attenuate the anisotropism of conduction along the different directions of cardiac muscle and thereby diminish the formation of reentry, a common mechanism underlying the formation of ventricular tachyarrhythmia [26]. However, optical mapping examination in the present study showed that the alcohol-treated mice had a slower conduction and more tachyarrhythmia. In the heart, the propagation of action potential across cardiomyocytes is via gap junctions. Although gap junctions provide low resistance pathways between adjacent cardiomyocytes, the conduction of gap-junctional channels is much slower compared to intracellular conduction. Therefore, when cells became smaller in size, for a defined dimension of myocardium, the propagation of action potential must pass more cells and gap junctions in between, and hence, required a longer time. In other words, the conduction became slower [27]. This (the smaller size of cardiomyocytes the slower conduction) suggested that, in the alcohol group of the present study, the change of conduction is attributable to the smaller cell size, which, together with lateralized gap junctions, also affects the anisotropism. Since slow conduction per se also favors the formation of reentry, this may explain that the alcoholic mice were prone to the occurrence of arrhythmia. In this view, increased expression and lateralization of the cardiomyocyte gap junctions may be a protective mechanism of the heart in response to the electric changes. Nevertheless, these findings are in agreement with the clinical observations that cardiac arrhythmias are frequently seen in patients with long-term heavy alcohol use [6].

## Conclusions

In conclusion, chronic 36% alcohol consumption induced profound remodeling of ventricular myocardium in mice, including change of the size of cardiomyocyte and the expression and distribution of Cx43 gap junctions, each of which may have distinct contribution to the electrical remodeling, characterized as slow down of conduction and occurrence of tachyarrhythmia.

## Methods

### Animals, alcohol administration, and preparation of hearts

The work was conducted in accordance with the Republic of China Animal Protection Law (Scientific Application of Animals), 1998. Eleven-week-old male C57BL/6 mice (purchased from National Laboratory Animal Center, Taipei, Taiwan) were given ad libitum normal chow (Purina 5001, LabDiet^®^, TestDiet company, Indiana, USA) and either double-distilled deionized water or 36% v/v alcohol as their only fluid source for 12 weeks. Thereafter, mice were anesthetized with pentobarbital (i.p., 60 mg/kg). The blood was collected (for determination of alcohol level using Ethanol-L3K Assay (Diagnostic Chemical Limited; Charlottetown, Prince Edward island, Canada)) and the hearts were taken out. The ascending aorta was cannulated and the heart was perfused with standard oxygenated Tyrode's solution (2.5 ml/min), paced (3-6 Hz, 4 ms duration, 3 fold threshold voltage) at the basal portion of the right ventricle from a stimulus electrode (S88J, GRASS, West Warwick, Rhode Island, USA), and stained with 20 μL of di-4-ANEPPS (Molecular Probe, Paisley, UK; 2 mM in DMSO). After the cardiac contraction was blocked with cytochalasin D (Sigma, Missouri, USA; 10 μM) the action potentials were recorded using optical mapping. Tiny needles were used to mechanically stabilize the heart. Fifty-eight murine hearts (29 in each group) were used for optical mapping. Conduction velocity, derived from activation map (Figure 5, lower panel), was calculated from the 9 mice of the alcohol group and compared to the initial 9 mice drivenable throughout the recording period of the control group.

In parallel, immediately after removal of the hearts, clearance of blood and weighing, the organs were either stored in liquid nitrogen for Western blotting and immunoconfocal microscopy, or prepared for histological examination and thin-section electron microscopy by standard procedures. In details, 14 hearts (7 in each group) were for Western blotting, 16 hearts (8 in each group) for immunohistochemical plus histology examination, and 6 hearts (3 in each group) for electron microscopy.

### Optical apparatus

Optical mapping was conducted using a stereo-fluorescence microscopy (Lecia MZ FLIII system; Heidelberg, Germany) with light (100-W high-pressure mercury vapor lamp; 106Z, Lecia) collimated and passed through a 470 nm interference filter, and focused on the frontal surface of left ventricle. Fluorescence emission from the stained heart was collected with a camera lens (45 mm, PLAN 1.0×, Lecia) through a 505-nm cut-off filter (0G515, Lecia) and focused to form an image on the surface of a 80 × 80 array CCD (CardioCCD39, RedShirtImaging, Georgia, USA). The image was magnified ×0.8 such that the CCD detected a 5 × 5 mm area of the epicardium. Spatial and temporal filtering were utilized in data post-processing. Isochronal maps of activation spread and conduction velocity measurements were derived using Cardioplex software (RedShirtImaging).

### Immunoconfocal microscopy

*Antibodies and detection systems *Rabbit polyclonal antisera (designated Cx43(R530)) against the synthetic peptides corresponding to 314 to 322 of the cytoplasmic C-terminal tail of rat Cx43 were used. The antisera were affinity-purified and have previously been confirmed to be isotype-specific [28]. Donkey anti-rabbit immunoglobulins conjugated to CY3 (Chemicon, California, USA) were used to visualize immunolabeled connexin. FITC-conjugated wheat germ agglutinin (WGA; Vector, Burlingame, California, USA) was used to detect the cell border. Anti-CD31 (BD Pharmingen, Franklin Lakes, USA) was used to detect the capillary. FITC-conjugated anti-rat immunoglobulins (Molecular Probes, Paisley, UK) were used to visualize immunolabeled capillary [29].

*Immunolabeling *Cryosections of the heart were fixed in -20*C methanol, rinsed in PBS for 5 minutes, blocked in 0.5% BSA (15 minutes), and incubated at 37*C with anti-Cx43 (1:300) for 1 hour or with anti-CD31 (1:200) for 2 hours. The samples were then treated with CY3-conjugated secondary antibody (1:500, room temperature, 1 hour). For single labelling of WGA, incubation was with WGA-FITC (1:500, 37*C, 1 hour). In double labeling experiments, incubation was with a mixture of anti-Cx43 (1:300) plus WGA-FITC (1:500) at 37*C for 1 hour, followed by incubation with the CY3-conjugated secondary antibody.

*Confocal laser scanning microscopy *Immunostained samples were examined by confocal laser scanning microscopy using a Leica TCS SP equipped with argon/krypton laser. Single WGA-FITC-labeled samples were used to measure the size of cardiomyocytes. For double labeling of Cx43 and WGA, the images were taken using simultaneous dual channel scanning. The images were collected using the × 40 objective lens and zoom 1.0 computer setting so that each pixel represented 0.23 μm. Each image recorded consisted of 1024 × 1024 pixels, and projection views of 4 consecutive optical sections taken at 1 μm intervals in the middle of the sections were recorded for analysis.

### Western blotting

Tissue samples of left ventricular free wall, ground to fine powders under liquid nitrogen, were lysed with 0.5 ml SB20 (containing 20% SDS, 0.1 M Tris pH 6.8, and 10 mM ETDA) and homogenized by sonication. After protein estimation, 2.5% 2-mercaptoethanol was added to the remaining samples. SDS-polyacrylamide gel electrophoresis was performed using minigels made of 4.5% stacking gels and 10% separation gels. Twenty μg of each sample, mixed with sample buffer (SB20 containing 2.5% 2-mercaptoethanol and 1% bromophenol blue) to make the final volume 15 μl, was loaded in each lane, subjected to electrophoresis (60 V for running in stacking gel and 120 V in separation gel; constant voltage), and transferred (20 V, constant voltage at room temperature overnight) onto PVDF membrane (Perkin Elmer, USA). The membrane was probed for Cx43 (1:200) and visualized using CDP-Star substrate solution (Roche, USA). Finally the membranes were stripped with 2% SDS, 0.7% 2-mercaptoethanol, and 0.1 M Tris pH 6.8, and probed with a mouse anti-β-actin antibody (1:5000; Chemicon).

### Analysis and statistics

Image analysis was conducted using QWIN image analysis software (Leica). The amount of capillary network was expressed as the number of capillary per unit area. For myocyte size and Cx43 gap junction distribution, images taken from the epicardial and endocardial portions of the sections at the transverse plane of left ventricle were used. The following data were obtained for each group: i) the length, width, and area of individual ventricular myocyte; ii) the total area of immunolabeled gap junctions per myocyte area, expressed in percentage; and iii) the percentage of immunolabeled gap junctions along the lateral borders of individual myocytes. For comparison of myocardial cellularity, cryosections stained with haematoxylin were recorded using a digital camera. The nuclei were counted and expressed as the number per unit area. For Western blotting, densitometric scanning and analysis were performed on the blots using Imagemaster (Amersham Pharmacia Biotech, New Jersey, USA). Results were expressed as mean ± SE. The data of histological examination and Western blotting were compared by Student's t test. For conduction velocity, data were compared by two way ANOVA with repeat measurement. Incidence of ventricular tachycardia, fibrillation were compared statistically by Chi square.

## Competing interests

The authors declare that they have no competing interests.

## Authors' contributions

YJ carried out the cardioelectrophysiologic studies, participated in the cardiac conduction, arrhythmia research and drafted the manuscript. CL carried out the morphometrical analysis of the study. RC participated in the electron microscopy. YM participated in the protein analysis (western blot and immunostaining). YS carried out the interpretation of the findings. CI participated in the design of the study and performed the statistical analysis. CH participated in the design of the study and critical comments of the manuscript. HI conceived of the study, and participated in its design and coordination and helped to draft the manuscript. All authors read and approved the final manuscript.
